# Serum Vitamin D Levels at Birth Are Not Associated With the Formation of Interglobular Dentin in Deciduous Teeth of Modern New Zealand Children

**DOI:** 10.1002/ajpa.70370

**Published:** 2026-10-16

**Authors:** Anne Marie Sohler‐Snoddy, Stephanie Chan, Rebecca Weastell, Victoria Chen, Benjamin J. Wheeler, Deanna Beckett, Carolina Loch

**Affiliations:** ^1^ Department of Biology University of North Carolina Greensboro Greensboro North Carolina USA; ^2^ Sir John Walsh Research Institute, Faculty of Dentistry, University of Otago Dunedin Otago New Zealand; ^3^ Department of Paediatrics and Child Health University of Otago Dunedin New Zealand

**Keywords:** dental development, mineral metabolism, paleopathology, vitamin D deficiency

## Abstract

Interglobular dentin (IGD) has been used as a biomarker for vitamin D deficiency (VDD) in past populations. However, an evidence‐based threshold for the levels of serum vitamin D associated with IGD occurrence has not been established. Here, we explore the association between perinatal serum vitamin D levels, presence of IGD, and biological sex in the deciduous teeth of modern children from Otago, New Zealand. Ground sections were created from the deciduous central or lateral mandibular incisors of 54 five to 6‐year‐old children (27 M, 27 F) with known perinatal blood vitamin D levels. Vitamin D levels at birth were classified as deficient (≤ 30 nmol/L), insufficient (> 30mn/L–< 50 nmol/L), or sufficient (≥ 50 nmol/L), and scanned slides were scored blind by two observers for IGD presence or absence. Thirty‐seven individuals (68.5%) exhibited IGD. The distribution of IGD was similar across vitamin D levels considered deficient (35.1%), insufficient (32.4%), and sufficient (32.4%), with no significant association between IGD presence and vitamin D status (*p* = 0.97) or IGD presence and sex (*p* = 0.77). Our findings do not support an association between IGD formation in deciduous teeth and vitamin D levels at birth. The prevalence of IGD across the sample, particularly in secondary dentin, suggests IGD may represent a normal developmental feature. However, it is important to note that cord blood vitamin D levels do not reflect the individual's vitamin D status during the entire period of deciduous incisor formation. Further longitudinal clinical research is required to better understand the relationship between VDD and the formation of IGD.

## Highlights

- Interglobular dentin (IGD) has been used as a proxy for poor vitamin D status in the past.
- An evidence‐based threshold for serum vitamin D levels associated with the formation of IGD is yet to be established.
- We explore IGD presence in the teeth of 54 children with known serum vitamin D levels at birth.
- We found no association between the presence of IGD and vitamin D status at birth or biological sex.

## 1 Introduction

Vitamin D is a prohormone produced following the action of UVB light on sterols in the skin and metabolized in the liver. Active vitamin D hormone (1,25 dihydroxyvitamin D; 1,25(OH)_2_ D) is produced by hydroxylation of vitamin D prohormone, primarily in the kidneys, although many types of cells express a vitamin D receptor and can create active vitamin D hormone locally (Bickle et al. 2019). 1,25(OH)_2_ D plays a key role in mineral metabolism, regulating the uptake of calcium from the intestine for uses in a wide range of physiological processes, including the mineralization of hard tissues like bone, dentin, and enamel (Bickle et al. 2019; Vautour and Goltzman 2019). Vitamin D deficiency is associated with many negative health outcomes but is most dramatically expressed in growing infants and young children as the metabolic bone disease rickets (Laurent et al. 2019; Munns et al. 2016). Rickets is a significant health problem in all socioeconomic categories (Cui et al. 2023; Munns et al. 2016). Global prevalence rates are difficult to determine, but a recent metanalysis of 308 studies from 81 countries found that 15.7% of individuals had serum vitamin D prohormone levels consistent with deficiency (Cui et al. 2023).

The macroscopic skeletal defects of vitamin D deficiency are well studied in the bioarchaeological record and have provided powerful insights into the human environment and behavior in the past (e.g., Mays et al. 2018; Veselka et al. 2019; Waters‐Rist and Hoogland 2018; see Brickley et al. 2020 for a review). However, recognition of rickets and adult osteomalacia requires identification of a suite of skeletal lesions. Poor skeletal preservation and completeness can inhibit the identification of this condition in the archeological record (Snoddy et al. 2016). Additionally, skeletal lesions related to vitamin D deficiency may remodel rapidly, particularly in infants and children, meaning that rickets and osteomalacia are often only identifiable in individuals who had active or recent disease at the time of death and are well preserved and complete (Brickley et al. 2020). The true scale of poor vitamin D status in any archeological cohort is therefore impossible to determine and macroscopically identified individuals must be considered “the tip of the epidemiological iceberg” (Snoddy et al. 2016).

Dental hard tissues, like bone, may be affected by disturbances in mineral metabolism during development, one possible manifestation being the formation of a microscopically visible structure known as interglobular dentin (IGD) (Nanci 2018; Seow et al. 1989). Dentin, which constitutes the bulk of the tooth's hard tissues, is formed incrementally by odontoblasts. These cells secrete a collagen‐rich organic matrix known as predentin, which subsequently mineralizes as odontoblasts retreat from the enamel‐dentin junction (EDJ) toward the pulp (Nanci 2018). Predentin mineralization occurs through the continuous deposition of hydroxyapatite crystals and may proceed via either globular or linear mineralization patterns (Nanci 2018). Globular mineralization occurs through the formation of spherical mineralization fronts known as calcospherites, which normally fuse to form a homogeneous mineralized tissue (Nanci 2018; Tjäderhane et al. 2009). Linear mineralization is a slower process characterized by a relatively uniform mineralization front without distinct mineralization foci (Nanci 2018). Globular mineralization predominates in rapidly forming crown dentin, whereas linear mineralization is commonly associated with the more slowly forming root dentin—although IGD may also be present in the roots (Sato et al. 2000). Interglobular dentin forms when calcospherites fail to fuse completely, leaving irregular hypomineralized or unmineralized regions within the circumpulpal dentin that appear histologically as scallop‐shaped spaces between areas of mineralized dentin (Jayawardena et al. 2009; Tjäderhane et al. 2009; Figure 1). Small, scattered areas of IGD immediately beneath the mantle dentin near the dentin‐enamel junction are considered a normal developmental feature and may reflect aspects of odontoblast organization and mantle dentin formation rather than pathological mineralization defects (Kagayama et al. 1997; Tsuchiya et al. 2002).

**FIGURE 1:**
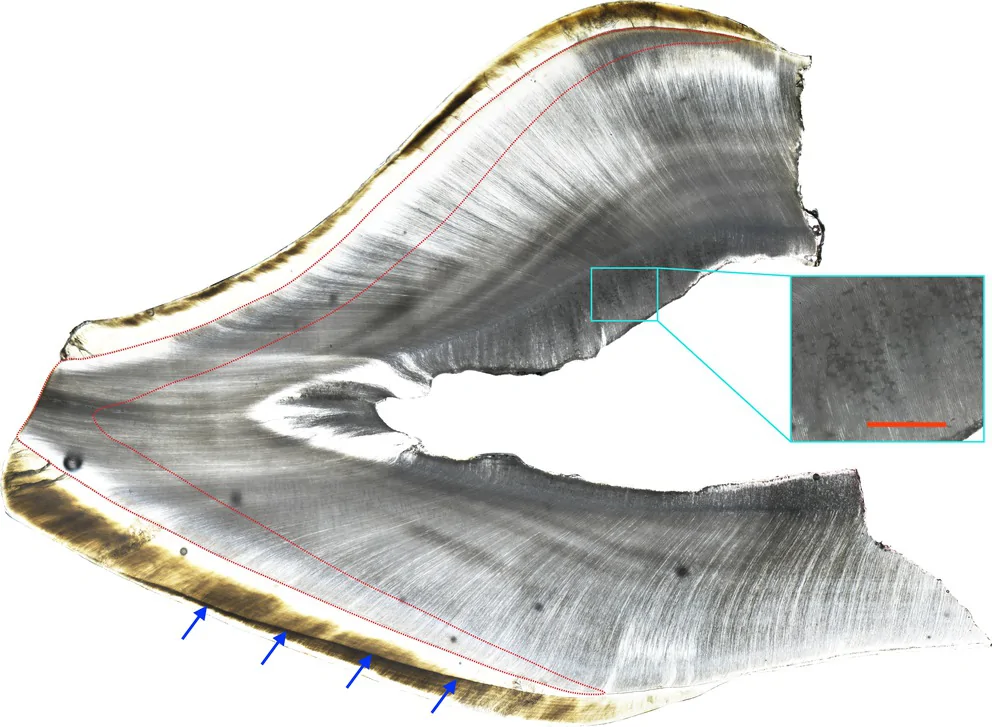
DC002, prenatally formed dentin is enclosed by the red dashed line and can be identified by its association with the neonatal line in the enamel (blue arrows). The serum vitamin D of this child at birth was 58.7 nm/L. Teal box shows IGD in postnatally formed dentin near the pulp chamber. Main image 40× total magnification, inset image 100× total magnification. Red bar = 200 μm.

In theory, any condition that limits the availability of calcium for hydroxyapatite production during dentin development could contribute to IGD formation including nutritional vitamin D deficiency, dietary calcium deficiency, and congenital metabolic defects of mineral metabolism. However, clinical evidence for pathological formation of IGD in humans comes primarily from children with X‐linked hypophosphatemic rickets rather than rickets from nutritional or other environmental factors (Abe et al. 1988; Boukpessi et al. 2006; Seow et al. 1989; Tracy et al. 1971; c.f. McDonnell et al. 1997). Past research with animal models has found IGD in the teeth of dogs with experimentally induced nutritional rickets (Mellanby 1928, 1931), and the vitamin D receptor (VDR) has been implicated in both enamel and dentin mineralization in mice (Zhang et al. 2009), although caution is needed with inferring human pathophysiological relationships from nonhuman experimental models. More recent bioarcheological research has uncovered evidence of IGD in individuals who have macroscopic skeletal lesions indicative of vitamin D deficiency (e.g., D'Ortenzio et al. 2016; Snoddy et al. 2024; Veselka et al. 2019), and IGD has been observed in the extracted teeth of a modern 2‐year‐old child with nutritional rickets (McDonnell et al. 1997). However, IGD is commonly seen in histological sections in modern human teeth (e.g., Jayawardena et al. 2009; Sato et al. 2000) and an evidence‐based threshold for serum vitamin D levels associated with the formation of IGD has not been established. Additionally, it is not known how intrinsic variables such as biological sex may influence the development of IGD. Given the increased use of IGD as a proxy for poor vitamin D status in archeological human populations, more work is needed to characterize the relationship between vitamin D status and IGD formation in individuals with known serum vitamin D levels and dentition forming during this time.

In this study we explore the relationship between serum vitamin D levels at birth and the presence of IGD in the deciduous incisors of a cohort of modern New Zealand children. We aimed to investigate whether serum vitamin D levels at birth are associated with IGD formation, and whether the presence of IGD varies among males and females.

## 2 Materials and Methods

Fifty‐four children (27 males, 27 females) were included in the current study. Samples from this cohort were previously collected as part of a larger longitudinal project (*Dental Consequences of Vitamin D Deficiency during Early Life*) following the health of children for whom serum vitamin D levels in cord blood at birth are known, alongside other biological and maternal factors (Beckett et al. 2023; Wheeler et al. 2018). Umbilical cord blood was originally collected from participants immediately following birth and serum vitamin D levels of each infant were determined from these samples (Wheeler et al. 2018). Serum total concentrations of 25(OH)D (sum of 25‐hydroxyvitamin D3 [25(OH)D3] and 25‐hydroxyvitamin D2 [25(OH)D2]) were measured by isotope‐dilution LC–tandem MS using an API 3200 instrument (Applied Biosystems) connected to a Dionex Ultimate 3000 HPLC system. Serum vitamin D status at birth was classified as deficient (≤ 30 nm/L), insufficient (> 30 nm/L–< 50 nm/L), and sufficient (≥ 50 nm/L) following the Global Consensus Recommendations of the Endocrine Society (Munns et al. 2016). Ethical approval for this work, which involved dental examinations, collection and histological analyzes of exfoliated teeth, was granted by the Otago University Human Ethics Committee (approval #H18/001). Written parental consent and child assent were obtained for all participants. Exfoliated deciduous central and lateral mandibular incisors were collected at dental appointments when the children were 6–7 years of age.

### 2.1 Sample Preparation

Teeth were embedded in epoxy resin (Epofix cold‐setting embedding resin, Struers, Copenhagen, Denmark) and sectioned along the central longitudinal axis of the tooth in a labio‐lingual direction with a Buehler IsoMet low speed saw with diamond wafer blade. The cut surface of the sample was hand‐polished using wet 600 grit silicon carbide paper and mounted to a labeled glass slide using Araldite. The mounted sample was then reduced to ~1 mm and sequentially ground using silicon carbide pads (320 & 600 grit) on a Buehler EcoMet Manual Grinder Polisher until a final thickness of 100–120 μm was reached. Ground specimens were then polished using a Buehler PSA backed MicroCloth with Buehler MicroPolish 0.3 μm alumina powder, sonicated to remove debris, dehydrated in 95% and 100% ethanol, and fixed with Histoclear.

### 2.2 Image Analysis

Samples were viewed under transmitted light on a Nikon Ni‐U microscope equipped with a DS‐Ri2 camera. NIS‐Elements D (v.5.10) was used to create a “virtual slide” at 40× total magnification with an additional region of interest (ROI) of the visible dentinal record captured at 100× total magnification.

Because existing methods for assessing IGD severity (e.g., D'Ortenzio et al. 2016; Snoddy et al. 2020) do not have a clinical foundation, here we chose to record only the presence or absence of IGD. IGD presence/absence was recorded: (1) within perinatally formed dentin (2) elsewhere in the pre or postnatal dentin. Perinatally formed dentin was identified by its positional association with the neonatal line in the enamel (Figure 1). Two researchers (VC and AS) examined all 40× and 100× scans and recorded presence or absence of IGD. This process was conducted with the examiners blind to the vitamin D status or other biological information of the donor child.

## 3 Results

A Cohen's Kappa test demonstrated moderate agreement between the more experienced observer (AS) and less experienced observer (VC) at the 95% confidence level (Kappa value 0.46). The two observers then examined all images together and reached a final consensus on IGD presence or absence for each sample. Thirty‐seven teeth (68.5%) exhibited evidence of IGD while 17 (31.5%) did not. When IGD was present, it was located on the periphery of the pulp chamber (*n* = 36), in the post‐natal secondary dentin, often demarcated by an abrupt change in the tubule orientation (Figure 1).

In this study, 19 samples (35.2%) were from children who were vitamin D deficient at birth, 18 (33.3%) were from children who were vitamin D insufficient, and 17 (31.5%) were children who were vitamin D sufficient at birth, representing a balanced distribution of vitamin D groups. Of the 37 samples with confirmed IGD presence, only 13 (35.1%) were from children who were vitamin D deficient at birth and none of these exhibited IGD in perinatally formed dentin. Twelve (32.4%) of the vitamin D insufficient children and 12 (32.4%) of vitamin D sufficient children also exhibited IGD. For the samples without evidence of IGD formation, there was an approximately even distribution of vitamin D status categories; 6 (35.3%) were from children who were vitamin D deficient at birth, 6 (35.3%) from children who were insufficient at birth, and 5 (29.4%) from children who were sufficient at birth. There was no significant association between vitamin D status at birth and presence of IGD (Pearson's chi squared test, *p* = 0.97). Similarly, there was no significant association between IGD occurrence and biological sex (Pearson's chi squared test, *p* = 0.77).

Results are outlined in Figure 2.

**FIGURE 2:**
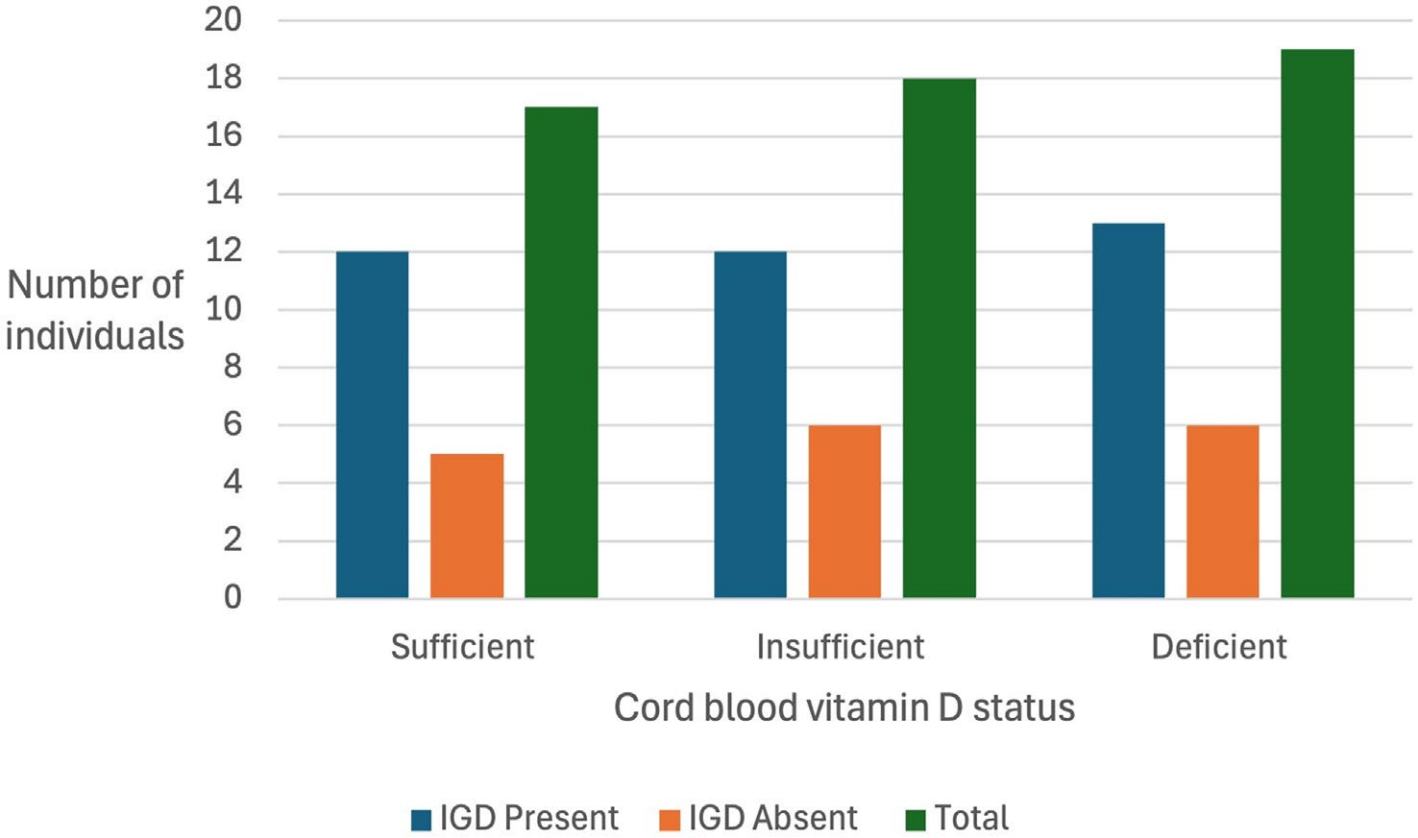
IGD presence/absence by vitamin D status at birth showing an approximately even distribution across all categories.

## 4 Discussion

This study evaluated the relationship between serum vitamin D status at birth and IGD formation in deciduous incisors, as well biological sex and IGD formation, in a cohort of modern human children. We failed to find a significant association between IGD and either of these variables. Interglobular dentin was nearly ubiquitous in our sample and had no relationship to serum vitamin D levels at birth. These findings are consistent with previous bioarcheological work showing IGD presence in 76% (16/25) of sampled teeth in individuals from an industrial era English cemetery (Snoddy et al. 2024), and 74% (37/50) of sampled individuals from 19th century Quebec (Bigué et al. 2024). Jayawardena et al. (2009) found IGD present in 90% (47/52) crowns of modern human teeth. Sato et al. (2000) found typical interglobular dentin present in the coronal dentin of most of the teeth analyzed, while the position and size of IGD was different among tooth types. The vitamin D status of the individuals in these studies is unknown, but their results support the experience of many clinical dental researchers—that IGD is a very common finding in human teeth.

Experimental animal models have demonstrated an association between vitamin D deficiency and interglobular dentin, suggesting a role for vitamin D deficiency in tooth mineralization regulation. Zhang et al. (2009) found that mice with vitamin D deficiency exhibited a reduction in early enamel hypomineralization and dentin mineralization, and in Mellanby's early 20th century studies, beagle dogs with a vitamin D deficient diet showed an increased frequency and prominence of enamel hypoplasia and IGD formation (Mellanby 1928, 1931). Building on these potential associations between IGD and vitamin D deficiency, as well as clinical papers on IGD in children with X‐linked hypophosphatemic rickets (Abe et al. 1988; Boukpessi et al. 2006; Seow et al. 1989; Tracy et al. 1971), recent bioarcheological publications have increasingly focused on using IGD as a biological marker for vitamin D deficiency in past populations (D'Ortenzio et al. 2016; Brickley et al. 2019; Bigué et al. 2024). However, to date, there is limited empirical evidence supporting an association between nutritional vitamin D deficiency during development and the formation of IGD. Here, we attempt to start addressing this knowledge gap. Below, we discuss our findings and raise questions to be tackled in future research.

### 4.1 Is the Prevalence of IGD Related to the Global Prevalence of VDD?

Vitamin D deficiency, defined by serum prohormone levels < 30 nm/L, is a widespread contemporary health concern (Munns et al. 2016). However, global data on the clinical prevalence of VDD is largely nonexistent, and primary studies of serum vitamin D status show wide regional variation. A recent metanalysis of 7.9 million individuals from 81 countries found that average global prevalence of VDD was 15.7%, but prevalence was highest in individuals living between 20° and 40° latitude (23.1%) and in individuals from lower‐middle income groups (26.7%) (Cui et al. 2023). Notably, these VDD prevalences are all far lower than the prevalence of IGD reported here and in other groups (Bigué et al. 2024; Jayawardena et al. 2009; Snoddy et al. 2024).

Notwithstanding the above, we did not find IGD present in the perinatally formed dentin of children who are *known* to have been vitamin D deficient at birth. Our findings do not support a relationship between vitamin D deficiency (according to the definition of the Endocrine Society Global consensus) and the formation of IGD in deciduous teeth. It is possible that the serum deficiency threshold required for the formation of what may be considered “pathological” IGD is substantially lower than the current clinical threshold for vitamin D deficiency. This needs to be explored in future clinical cohort studies. However, current evidence does not support the hypothesis that the high prevalence of IGD found here and elsewhere is associated with nutritional vitamin D deficiency.

### 4.2 Is Location, “Severity,” and Periodicity of IGD Important?

As discussed above, the strongest clinical evidence for a connection between IGD and defective mineral metabolism comes from children with hereditary rickets, particularly familial X‐linked hypophosphatemia (e.g., Abe et al. 1988; Boukpessi et al. 2006). X‐linked hypophosphatemic rickets is caused by a mutation on the PHEX gene, which regulates phosphate homeostasis and is expressed in both osteoblast and odontoblast cells (Boukpessi et al. 2006). Previous clinical work found that IGD is far more pronounced in the mantle and primary circumpulpal dentin of children with X‐linked hypophosphatemic rickets, although its severity is still variable both within and between the teeth of these children (Abe et al. 1988; Boukpessi et al. 2006; Clayton et al. 2021; Tracy et al. 1971). The clinical signs of rickets and osteomalacia always result from relative hypophosphatemia, but there are many underlying causes that can result in low serum phosphate, including other inborn metabolic errors (e.g., vitamin D dependant or resistant rickets), acquired kidney or liver disease, or exposure to environmental factors that disrupt mineral metabolism (Laurent et al. 2019). “Nutritional” rickets can take either vitamin D deficiency or calcium deficiency predominant forms, and results from poor vitamin D pro‐hormone synthesis due to low UVB exposure or poor dietary calcium uptake, respectively (Laurent et al. 2019; Munns et al. 2016; Vlok et al. 2023). These “nutritional” factors are by far the most common etiologies of rickets and osteomalacia, but the only available studies of IGD in cases of presumed nutritional rickets in humans are from anonymous archeological populations (e.g., Bigué et al. 2024; Cockerille et al. 2026; Colombo et al. 2018; Snoddy et al. 2024; Veselka et al. 2019). These are individuals for whom serum vitamin D status during development cannot be established, for obvious reasons.

A potential explanation for our findings is that IGD in deciduous teeth could form at much lower serum vitamin D levels than the those exhibited by vitamin D deficient children in our cohort (the lowest serum vitamin D level in our cohort was 14.2 nM/L). If this is true, are IGD location, subjective “severity,” and periodicity important when considering IGD as a possible marker of impaired mineral metabolism? We have demonstrated that IGD in the secondary dentin of deciduous teeth is a common finding and argue that it may be related to structural differences in this tissue. However, published evidence for “severe” episodic occurrences of IGD in primary dentin in archeological human teeth is prolific (e.g., Bigué et al. 2024; Brickley et al. 2019; D'Ortenzio et al. 2016; Snoddy et al. 2024). In some of these instances, IGD appears to form in approximately annual increments, which could suggest seasonal vitamin D deficiency (Snoddy et al. 2024; Veselka et al. 2019), particularly when the environmental context in which these individuals lived is considered.

### 4.3 Primary Versus Nonprimary Dentin and IGD

An intriguing finding of this study was the unexpected location of IGD in secondary dentin. The bulk of the tooth is made up of primary dentin, which is laid down during dental formation prior to the teeth reaching occlusion (Nanci 2018). IGD caused by abnormalities in mineral metabolism during dentin development (e.g., during the perinatal and infant period) should therefore be present in primary dentin. Secondary dentin forms after the tooth has erupted and is in occlusion and generally increases in abundance with age due to the accumulative effects of masticatory stress (Murray et al. 2002; Nanci 2018). Secondary dentin is less well‐studied in deciduous teeth and there has been some confusion with its nomenclature. Klinge (1999, 2001) has termed the phenomenon we describe here in deciduous teeth as “tertiary dentin.” However, tertiary dentin or reparative/reactionary dentin, forms in response to injury or disease, reenforcing discreet areas that are structurally compromised (e.g., where a carious lesion or fracture is present) (Nanci 2018). We believe that the uniform deposition of additional dentin around the pulp cavity, with or without IGD voids, in deciduous teeth is secondary dentin deposition and likely forms in response to masticatory stress. Nearly all the incisors in our sample exhibited heavy attrition with dentin exposure, which supports this.

In their study of non‐primary dentin in 20 modern deciduous teeth via SEM imaging, Klinge found that “tertiary” dentin exhibited fewer tubules and was less mineralized than primary dentin (Klinge 1999). Light microscopy of ground sections of these teeth revealed an abundance of interglobular dentin in these regions as well. In a later study utilizing both ground, undecalcified teeth and decalcified thin sections, Klinge found that the pre‐dentin matrix in these regions is fundamentally different to that of primary dentin, being denser and more fibrous (Klinge 2001). More work is needed to characterize nonprimary dentin in deciduous teeth, but these findings suggest that our observations of IGD in this sample are part of “normal” secondary dentin formation in response to masticatory stress rather than the result of disturbances to systemic mineral metabolism. For now, we recommend that IGD in these regions be considered a developmental feature rather than a “true” mineralization defect.

## 5 Conclusions and Limitations

This study is the first attempt to analyze IGD in children's teeth with known vitamin D levels. We have demonstrated that clinical vitamin D deficiency by cord blood status is not associated with the formation of IGD in the dentin of deciduous incisors. Pending further clinical research clarifying the relationship between IGD and vitamin D status, it may be prudent for paleopathologists and bioarcheologists to interpret this feature in a manner similar to periosteal new bone formation (Weston 2012). That is, it is a common finding in human remains, its underlying causes are multiple and include normal development, and the location, degree of expression, and social and environmental context in which the individual lived are key factors in differential diagnosis.

One of the key limitations with this study is its cross‐sectional nature—our data is limited to the brief perinatal period and does not cover periods of the life‐course known to be associated with increased risk of metabolic bone disease (i.e., weaning, infancy). Future studies with larger samples sizes and longitudinal designs could provide more definitive insights into the potential influence of vitamin D levels on IGD formation throughout growth and development. In the meantime, we encourage bioarcheologists to consider this feature with caution when exploring vitamin D deficiency in past populations.

## Author Contributions

**Anne Marie Sohler‐Snoddy:** conceptualization, investigation, writing – original draft, methodology, formal analysis, supervision, resources. **Stephanie Chan:** writing – review and editing, investigation, formal analysis. **Rebecca Weastell:** investigation, writing – review and editing, formal analysis. **Victoria Chen:** investigation, writing – review and editing, formal analysis. **Benjamin J. Wheeler:** writing – review and editing, data curation, project administration. **Deanna Beckett:** data curation, resources, writing – review and editing, investigation, project administration. **Carolina Loch:** resources, supervision, data curation, project administration, writing – review and editing, investigation, conceptualization, methodology.

## Ethics Statement

Ethical approval was granted by the Otago Human Ethics Committee (approval #H18/001).

## Supporting information


**Data S1:** ajpa70370‐sup‐0001‐DataS1.xlsx.

## Data Availability

The data that supports the findings of this study are available in the Supplementary Material of this article.
